# Application of a phenotypic drug discovery strategy to identify biological and chemical starting points for inhibition of TSLP production in lung epithelial cells

**DOI:** 10.1371/journal.pone.0189247

**Published:** 2018-01-10

**Authors:** Adelina Orellana, Vicente García-González, Rosa López, Sonia Pascual-Guiral, Estrella Lozoya, Julia Díaz, Daniel Casals, Antolín Barrena, Stephane Paris, Miriam Andrés, Victor Segarra, Dolors Vilella, Rajneesh Malhotra, Paul Eastwood, Anna Planagumà, Montserrat Miralpeix, Arsenio Nueda

**Affiliations:** Almirall R&D Center, Almirall S.A., Sant Feliu de Llobregat, Barcelona, Spain; Universita degli Studi di Bologna, ITALY

## Abstract

Thymic stromal lymphopoietin (TSLP) is a cytokine released by human lung epithelium in response to external insult. Considered as a master switch in T helper 2 lymphocyte (Th2) mediated responses, TSLP is believed to play a key role in allergic diseases including asthma. The aim of this study was to use a phenotypic approach to identify new biological and chemical starting points for inhibition of TSLP production in human bronchial epithelial cells (NHBE), with the objective of reducing Th2-mediated airway inflammation. To this end, a phenotypic screen was performed using poly I:C / IL-4 stimulated NHBE cells interrogated with a 44,974 compound library. As a result, 85 hits which downregulated TSLP protein and mRNA levels were identified and a representative subset of 7 hits was selected for further characterization. These molecules inhibited the activity of several members of the MAPK, PI3K and tyrosine kinase families and some of them have been reported as modulators of cellular phenotypic endpoints like cell-cell contacts, microtubule polymerization and caspase activation. Characterization of the biological profile of the hits suggested that mTOR could be a key activity involved in the regulation of TSLP production in NHBE cells. Among other targeted kinases, inhibition of p38 MAPK and JAK kinases showed different degrees of correlation with TSLP downregulation, while Syk kinase did not seem to be related. Overall, inhibition of TSLP production by the selected hits, rather than resulting from inhibition of single isolated targets, appeared to be due to a combination of activities with different levels of relevance. Finally, a hit expansion exercise yielded additional active compounds that could be amenable to further optimization, providing an opportunity to dissociate TSLP inhibition from other non-desired activities. This study illustrates the potential of phenotypic drug discovery to complement target based approaches by providing new chemistry and biology leads.

## Introduction

Thymic stromal lymphopoietin (TSLP) is an epithelial and mast cell-derived cytokine linked to allergic diseases such as asthma and atopic dermatitis (AD). In addition to its pro-inflammatory activity, TSLP appears to play a homeostatic role in tissues like the gut where it has been related with the blockade of T helper 1 lymphocyte (Th1)/Th17 responses. TSLP has also been involved in the biology of certain types of cancer, where its role is less clear and appears to be context dependent [1, 2].

TSLP is highly expressed in human cutaneous epithelial cells in AD and bronchial epithelial cells in asthma [3, 4] and is believed to participate in the progression from severe AD to asthma and allergic rhinitis (“atopic march”) [5, 6]. TSLP plays a role during the sensitization/priming phase of innate and adaptive allergic responses, inducing cytokine production in mast cells and immature dendritic cells (DCs), and stimulating mature DCs to prime CD4+ Th2 cells. During the challenge phase, TSLP supports Th2 CD4+T-cell proliferation and also induces cytokine production [7, 8]. TSLP expression is increased in the airway epithelium and lamina propria of patients with severe asthma even during high-dose inhaled or oral corticosteroid therapy, suggesting the potential of anti-TSLP treatment in corticosteroid resistant severe asthma with increased Th2 inflammation [9]. A short and a long isoform of TLSP have been identified which have distinct gene promoters that are differentially controlled by external stimuli [2]. The short isoform is constitutively expressed in several tissues and has been linked to TSLP homeostatic functions, while expression of the long isoform is inducible and appears to correlate with pathologies like asthma, AD or psoriasis [2]. The long isoform of TSLP can be upregulated by TLR-3 dependent polyinosinic:polycytidylic acid (poly I:C) stimulation in human bronchial epithelial cells (NHBE) [10, 11].

Systems-based phenotypic drug discovery can provide new unprecedented opportunities to complement the well proven target based strategies [12, 13, 14, 15, 16]. Even for target based approaches, the use of a phenotypic assay in the form of a cellular functional model with a target dependent phenotypic endpoint offers the possibility to access the most relevant target variant or molecular mechanism of action without any previous bias [12, 17]. Phenotypic drug discovery strategies have delivered several new drugs and drug candidates in recent years [18, 19, 20, 21, 22, 23] and are increasingly being used to complement the target based approach.

In this study, starting from a target based concept focused on the inhibition of TLSP function as an immune mediator, a phenotypic screening system was selected which recapitulated virus / allergen-induced TSLP production in primary human bronchial epithelial cells. We interrogated this system with our chemical library to identify novel chemical starting points for optimization and new potential mechanisms of action mediating the production of TSLP (“biological starting points”).

## Materials and methods

### Human primary cells, cell lines and cell culture conditions

Normal human bronchial epithelial cells (NHBE) (Lonza CC-2540,) corresponding to 2 different lots derived from 2 human healthy donors, henceforth referred to as donors #1 and #2, were purchased from Lonza, who provided the cells after obtaining permission for use in research applications by informed consent. Cells were cultured in 5% CO_2_ at 37°C according to the manufacturer´s instructions using Lonza´s Bronchial Epithelial Basal Medium (BEBM) (CC-3171) supplemented with Lonza´s Bronchial Epithelial Cell Growth Medium bullet kit (BEGM) (CC-4175), and grown in collagen coated T-75 and T-175 tissue culture flasks as required (Becton Dickinson 356485 and 356487). Subculturing after trypsinization and amplification was performed using Lonza´s reagent pack subculture reagents (CC-5034) following the manufacturer´s instructions. CHO-K1 and THP-1 cells were purchased from the ATCC through LGC standards (ATCC CCL-61 and TIB-202 respectively). The LAD2 cell line is a human mast cell line established at the National Institutes of Health (Bethesda, Maryland, U.S.) [24].

### Compound preparation, plate selection and *in silico* predictions

For HTRF assays, test compounds were resuspended in DMSO (Scharlab SU01571000,) at a concentration of 1.33x10^-3^ M, shaken for 10 minutes and diluted 1/50 in BEBM medium containing 1x ITS supplements (insulin, transferrin and selenium) (Lonza 18-838Z) and 0.1% BSA (bovine serum albumin) (Sigma A3059). 1 μl of this stock solution was used directly for the primary HTS and confirmations assays resulting in a final concentration of 1.06 μM of test compound and 0.1% DMSO. For dose response curves determinations, stocks of test compounds at a concentration of 1.33x10^-3^ M in DMSO were sequentially diluted at 1:4 dilution steps before further 1/50 dilution in BEBM medium containing 1x ITS and 0.1% BSA. For the highest 4 μM concentration test compounds were resuspended in DMSO at a concentration of 2.5x10^-3^ M and diluted 1/25 in BEBM medium containing 1x ITS and 0.1% BSA before adding 1 μl to the wells containing the cells.

A selection of 144 plates from the Almirall compound library was tested in the primary screen, corresponding to 44,974 compounds. These plates were selected based on the following criteria: physico-chemical properties compatible with crossing a cellular membrane (molecular weight <400, LogD <4, polar surface area between 70 and 140), lack of structural alerts (reactive and toxic groups), predicted low cytotoxicity (Bayesian model to predict cytotoxicity based on historical results stored in our database) and diversity, defined as the total number of assemblies divided by the number of molecules (scaffold enrichment) and number of structural clusters / plate: based on the fingerprint features (the more clusters per plate the better). *In silico* predictions for potential targets of compound **1** were performed using CT-Link software (Chemotargets) [25].

As internal controls for the HTRF assay, a dose response curve of standard compound BX795 (InvivoGen tlrl-bx7) [26] was included in each plate. Reference compounds everolimus [27] and AZD8055 [28] were purchased from AK Scientific. Tofacitinib [29] was purchased from MedChem Expres. Compound **1** was obtained from Asinex, ouabain [30] from Tocris, nocodazole [31] from Prestwick and compound **6** [32] from Tripos. Compound **15** was purchased from SIGMA and compound **16** from Bionet. All other compounds were synthesized in the context of their respective programs as indicated, and were part of the Almirall compound collection. Chemical structure of the hits was confirmed by UPLC/MS.

### TSLP HTRF assay

For homogeneous time resolved fluorescence (HTRF) assays, cells were amplified for 13–15 days with 2 sequential passages of amplification at 4 and 9 days after thawing from the original vial. Cells were grown to confluency, processed using standard trypsinization, plated at 25,000 cells/well in collagen coated 384 well plates (BD Biosciences 356664) in 20 μl of BEGM supplemented BEBM medium using a Multidrop^™^ (Thermo Fisher Scientific) and left overnight for 20–24 hours under standard cell culture conditions (37°C, 5% CO_2_, 90% humidity) in a Cytomat^™^ incubator (Thermo Fisher Scientific). All subsequent steps were automated using a Sciclone^®^ G3 workstation (Perkin Elmer) with a Twister^™^ II robot (Perkin Elmer). After incubation, cells were washed with Hepes buffered saline 1x solution (BSS) (Lonza CC-5024) and resuspended in 20 μl of BEBM medium containing 0.1% BSA (Sigma A3059-100G) and 1x ITS. 1 μl of test compound solution prepared at 25x concentration was added to the wells containing NHBE cells. The plates were then incubated during 90 minutes at 37°C, 5% CO_2_, 90% humidity before adding poly I:C (Invivogen HMW Tirl-pic-5) and human IL-4 (Peprotech 200–04) stimulus in 4 μl of BEBM medium containing 1x ITS, 0.1% BSA and 0.1% DMSO using a Multidrop^™^. This resulted in a final concentration of 50 μg/mL poly I:C, 50 ng/mL IL-4 and 0.1% DMSO. Cells were then incubated for 24h in the presence of test compounds and stimulus at 37°C, 5% CO_2_ and 90% humidity conditions. After the 24h incubation period the plates were transferred to a CyBi^®^-Well platform (CyBio AG) to collect 10 μl of the supernatants which were transferred to a new plate. TSLP levels in the supernatant were determined using a homogeneous Time-Resolved Fluorescence (HTRF) assay custom developed by Cisbio. The assay was based in the use of a donor dye (europium cryptate) coupled to a monoclonal anti-TSLP antibody and an acceptor dye (d2) coupled to a second polyclonal anti-TSLP antibody. Interaction of the two anti-TSLP antibodies with secreted TSLP protein brings the dyes into close proximity with each other and specific wavelength (665 nm) fluorescence can be detected which is proportional to the amount of TSLP in the supernatant. The assay was run at room temperature in solid white, nonbinding surface 384-well plates (Corning 3673). Plates were read in an Envision^®^ plate reader (Perkin Elmer) at room temperature using the HTRF mode with the laser light source for excitation and 665 nm and 620 nm for emission after overnight incubation of the samples with antibody conjugates. To test the possible interference of the compounds with the HTRF technology, 1 μl/well of the 1:50 dilution of the compounds was transferred to a solution containing 177 pg/ml of recombinant TSLP (R&D Systems 1398-TS-010) in BEBM medium containing 1x ITS, 0.1% BSA. 10 μl of the resulting solution was then used to determine TSLP levels as described above.

### Real-time and endpoint PCR

For real-time PCR (qPCR) and endpoint PCR, cells from donor #1 were amplified and harvested as described for the HTRF assay. Following trypsinization, 10^5^ cells/well were plated in collagen coated 96 well plates (BD Biosciences 354650) in 100 μl of BEGM supplemented BEBM medium. Cells were incubated overnight at 37°C and 5%CO_2_. The next day, the medium was removed and replaced with ITS/BSA medium supplemented with antibiotics. Test compounds were added at a final concentration of 4 μM and pre-incubated in the same conditions than for TSLP HTRF assays, 60 minutes previous to stimulation with poly I:C and IL-4 at 50 μg/mL (10 μg/mL for endpoint PCR) and 50 ng/mL respectively. 6 hours after stimulation and compound incubation the medium was removed and cells were washed and conserved at -80°C until RNA extraction. RNA extraction was conducted using the RNeasy^®^ 96 kit (Qiagen RNeasy^®^ 96 kit, 74181). For each 96 well plate, cells were lysed on-plate and treated with ethanol. Cell lysates were loaded into the wells of the RNeasy^®^ 96 plate and RNA bound to the filter was washed three times and eluted with RNase-free water. For each well containing 10^5^ cells, total RNA was recovered in 100 μL of RNase-free water. Purified RNA was stored at -80°C. For synthesis of cDNA and qPCR, 2 qPCR reactions were performed using a TSLP (Hs01572933_m1) (FAM|L: 2900) or a GAPDH (Hs03929097_g1) (VIC_PL– 2900) TaqMan^™^ probe (Thermo Fisher Scientific). The reaction was performed with 5 μL of RNA per well using the iScript^™^ One-Step RT-PCR kit for probes (Bio-Rad 170–8895) and a Bio-Rad CF-X 96 Real Time System. The average Ct ± SD for GADPH in DMSO treated samples was 27.47 ± 0.39 (n = 32). In compound treated samples, the average Ct ± SD for GADPH was 27.50 ± 0.51 (replicate 1) and 27.47 ± 0.51 (replicate 2) (n = 121). For endpoint PCR, the following primers were used: GADPH forward (GGG GAG CCA AAA GGG TCA TCA TCT), GADPH reverse (GAG GGG CCA TCC ACA GTC TTC T), TSLP forward (GAG TGG GAC C AAA AGT ACC G) and TSLP reverse (TGG GCA CCA GAT AGC TAA GG). GADPH primers were designed to amplify a 235 base pair (bp) fragment. TSLP primers were designed to amplify a 168 bp fragment corresponding to the long inducible form of TSLP [10]. All primers were synthesized at SIGMA. cDNA synthesis was performed with Clontech´s Advantage^®^ RT-for-PCR Kit (639506) using 1 μg of RNA in 20 μl of reaction volume. The final product was diluted adding 80 μl of RNase-free water and stored at -80°C. Endpoint PCR reactions were performed in a total volume of 25 μl with 2.5 μl of the cDNA, 0.5 μl (0.5 U) of SIGMA Taq DNA Polymerase (D-6677), 200 μM of each dNTP (SIGMA DNTP10 Set), 1x SIGMA PCR buffer (P-2192) and 0.5 μM each of the corresponding GADPH or TSLP primers. Cycling conditions were as follows: 95°C incubation for 10 minutes followed by 42 cycles at 95°C / 30 seconds, 55°C / 30 seconds, 72°C / 30 seconds and a final 72°C incubation for 5 minutes. The reaction was performed using a G-Storm GS1 Thermal Cycler (Gene Technologies), and analyzed using 1.5% agarose gel electrophoresis and a Bio-Rad ChemiDoc XRS+ imaging system. The reference marker used was 100 bp DNA ladder from VWR (K180).

### Cell viability and additional biochemical and cellular assays

The viability of NHBE and CHO-K1 cells was quantified using ATP as an indirect read out of the potential cytotoxicity of the compounds. For NHBE, after supernatants were collected from the cell plates, cells were washed, 20 μl of DPBS (Sigma D8537) were added and the cells were processed using the ATPlite^™^ 1step luminescence based assay (PerkinElmer 6016731) following the manufacturer´s instructions, using 20 μl of ATPlite^™^ reagent to quantify the amount of ATP in the sample. Plates were shaken and covered with aluminium foil for 25 min. Luminescence was measured in an Envision reader (Perkin Elmer). Evaluation of viability in CHO-K1 cells was used as an internal compound cytotoxicity standard across cellular assays. For CHO-K1 viability assays in 96 well format, 10,000 cells/well were plated in white 96-well plates (Perkin Elmer 6005181) in F12 medium (Sigma F-6658) with 10% of BioWhittaker fetal bovine serum (FBS, Lonza, DE14-801F) and supplemented with 1% Gibco^®^ glutamine (Thermo Fisher Scientific 25030). The plates were incubated for 24 h at 37°C in 5% CO_2_. After the incubation period, the culture medium was removed and washed with 100 μl of DPBS. Test compounds were dissolved and serially diluted in 100% DMSO. A final 1/100 dilution of each concentration was performed using F12 culture medium with 0.5% FBS. A volume of 100 μl culture medium containing the compounds or 1% DMSO (for control wells) was added to the wells containing the cells and the plate was incubated again 24h. After 24h of compound exposure, cells were washed with DPBS. Then, 100 μl of PBS were added, followed by the addition of 100 μl of ATPlite^™^ reagent. The plate was shaken for 25 minutes using a microtiter plate shaker. Luminescence was measured using a Luminoskan Ascent luminometer (Thermo Fisher Scientific). For Everolimus and AZD8055, the CHO-K1 viability assay was performed in 384 well plates (Perkin Elmer 6007680), using 2,500 cells / well in 40 μL of incubation medium containing the test compounds and adding 40 μL of ATPlite reagent directly to the cells before measuring the luminescence.

Quantification of enzymatic p38 activity and p38-dependent TNF production in THP-1 cells was performed as previously described [33]. Enzymatic Syk kinase activity assays and Syk dependent LAD2 cells degranulation assays were performed as previously described [34]. PI3Kδ dependent pAkt phosphorylation in macrophage colony-stimulating factor (M-CSF) stimulated THP-1 cells was measured as previously described [35].

For determination of JAK3 kinase biochemical activity, the compounds were added to a mixture containing 0.6 μM ATP (Sigma A7699) and poly GT-Biotin (Cisbio 61T66KLA) (140 nM). The catalytic reaction was then started with the addition of JAK3 recombinant enzymes (Carna Biosciences 04–046) to a final concentration of 0.3 nM. The reaction was incubated during 30 minutes and a mixture of the Streptavidin-XL65 (Cisbio 61T66SAXLA) and Phospho-Tyrosine Cryptate antibody (Cisbio 61T66KLA) was then added to stop the reaction according to the manufacturer´s instructions. Cellular JAK1/3 activity was evaluated using the CellSensor^®^ STAT6-bla RA-1 cells, a Ramos cell line derivative containing a beta-lactamase reporter gene under control of the STAT6 response element (Thermo Fisher Scientific K1646). For the assay, 30,000 cells / well were plated in a 384-well plate (Corning 3712) and cultured overnight at 37°C with 5% CO_2_ in Gibco Opti-MEM^®^ media (Thermo Fisher Scientific 11058–021) containing 556 μg/mL CD40 (Thermo Fisher Scientific PHP0025), 0.5% heat inactivated FBS (Thermo Fisher Scientific 10082–147), 10 μM non-essential amino acids (Sigma M7145), 100 μM Sodium Pyruvate (Sigma 11360–039) and 100 U/mL Penicillin (Sigma P0781). Test compounds and hIL-4 (PrepoTech 200–04) at its EC_80_ were incubated 4h at 37°C with 5% CO_2_ in a final concentration of 0.5% DMSO. After incubation, 8 μl of detection reagent (Thermo Fisher Scientific K1138) were added and the mixture was incubated in the dark for 2 additional hours. Finally, the FRET signal was measured in an Envision reader (Perkin Elmer).

### Selectivity panels and bioactivity profiling

The concept of the BioMAP^®^ Diversity PLUS^™^ Platform^™^ (DiscoverX) has been previously described [36, 37]. The platform is comprised of 12 specific combinations of human primary cell culture and co-culture systems stimulated with a range of physiological and disease relevant stimuli (S2 Table). The resulting activities define a bioactivity fingerprint that was used to search for similar patterns of biological responses across the BioMAP^®^ reference database of >3,000 compounds, biologics, approved drugs and experimental agents. Compound **4** was tested at 0.111 μM, 0.333 μM, 1 μM and 3 μM concentrations.

Kinase selectivity was evaluated at Eurofins Pharma Discovery Services using a selection of 125 representative kinases tested at a concentration of 10 μM in duplicate (S3 Table). IC_50_ values for selected activities were further characterized at 9 duplicate concentrations. DNA-PK activity was determined at the SelectScreen^®^ Biochemical kinase Profiling Service of Thermo Fisher Scientific.

General selectivity profiling was performed at Eurofins Pharma Discovery Services using the BioPrint^®^ panel and reference database. The panel included 104 binding assays (non-peptide, peptide and nuclear receptors, ion channels and amine transporters) and 41 cell based and enzyme assays (including 10 kinases, 9 proteases and 5 phosphodiesterases) (S4 Table). More than 70% were human targets. Compound **4** was tested at a concentration of 1 μM in duplicate (10 μM for P-gp and CYP enzymes). The resulting bioactivity fingerprint was compared with a database of >2,500 BioPrint marketed drugs and reference compounds. IC_50_ values for selected activities were further characterized at 8 concentrations in duplicate.

### Calculations

Data calculations were performed using Excel fit software and dose-response curves were adjusted using equation 203 (four-parameter logistic equation). To calculate TSLP levels in the samples, a standard curve was performed in a separate plate using recombinant TSLP (1398-TS-010) (R&D Systems) prepared in 1% BSA in DPBS (Sigma D8537). The standard curve was built with eight points prepared in ITS/BSA in BEBM medium without supplements, starting at 700 pg/ml and diluted 1:2, with medium alone being zero TSLP. Fitting model corresponded to equation 100 of Excel fit software. Data from the primary phenotypic screen was visualized using Vortex (Dotmatics). Primary screening was performed in single point at a concentration of 1.06 μM. The confirmation screen was performed in duplicate also at a concentration of 1.06 μM. For compound selection the maximum inhibition of the two individual duplicate values was taken into account. Dose response confirmation was performed at single point in the 2 donors tested. 9 point curves were run starting at 4 μM concentration by doing serial 1:4 dilutions in cells derived from both donors.

In the qPCR assay the TSLP ΔCt of each sample was calculated with respect to the Ct of GADPH in the same sample. The % of inhibition of TSLP mRNA levels in samples treated with test compounds was calculated as a ΔΔCt with respect to the total inhibition obtained in samples treated with the standard compound BX795. The calculated %I was obtained from two biological replicas in the same experiment. For hit validation, the % of inhibition of TSLP mRNA levels calculated by qPCR was correlated with the percentage of inhibition of TSLP protein production (measured by HTRF).

For CHO-K1 viability assays, a standard curve of cells was included in the plate. Luminescence values versus cell number were plotted and the data adjusted to a linear equation. Luminescence values of the samples were interpolated to obtain number of cells. IC_50_ values were obtained by non-linear regression using Activity Base software (IDBS) and a four-parameter log equation.

## Results

### Optimization of phenotypic assay

A phenotypic assay to capture inhibitors of TSLP production in human primary bronchial epithelial cells using a 384 well plate format was set up and optimized in order to screen the selected compound library. A combination of poly I:C and IL-4 was selected as a relevant stimulus recapitulating a virus / allergen mediated insult [38]. Simultaneous activation with these two stimuli synergistically upregulates TSLP production in NHBE cells to higher levels than separate stimulation by poly I:C or IL-4 alone [38]. The production of TSLP under these conditions was verified in NHBE cells from the 2 selected donors and resulted in 50–60 fold induction of TSLP protein levels (Table 1). Analysis by endpoint PCR confirmed the presence of mRNA corresponding to the long inducible isoform of TSLP in NHBE cells from donor #1 after poly I:C / IL-4 stimulation (S1 Fig).

**Table 1:** Production of TSLP in NHBE cells from the 2 donors used in this study.

Donor	TSLP levels without stimulation (pg/mL)	TSLP levels after 24h stimulation (pg/mL) ^a^
#1	16 ± 14	952 ± 84
#2	20 ± 14	1,033 ± 384

Results shown as mean ± standard deviation (from 2 individual experiments with n = 5 determinations each).

^a^ Cells stimulated with 50 μg/mL of poly I:C and 50 ng/mL of human IL-4.

Additional parameters were fine tuned in order to achieve adequate throughput and sufficiently robust assay conditions: media and buffer, plate type, biological stimulus, cell density, compound pre-incubation time, DMSO concentration, stimulation time and automation parameters. Final assay conditions are summarized in Table 2.

**Table 2:** Summary of final conditions used for the phenotypic assay.

Condition	Value
Cell number (cells / well)	25,000
Compound pre-incubation time (h)	1.5
Stimuli	50 μg/mL poly I:C + 50 ng/mL IL-4
Stimulation time (h) (in the presence of test compounds)	24
Assay buffer	ITS / BSA in BEBM medium without supplements
Final DMSO concentration (%)	0.1

**Fig 1:**
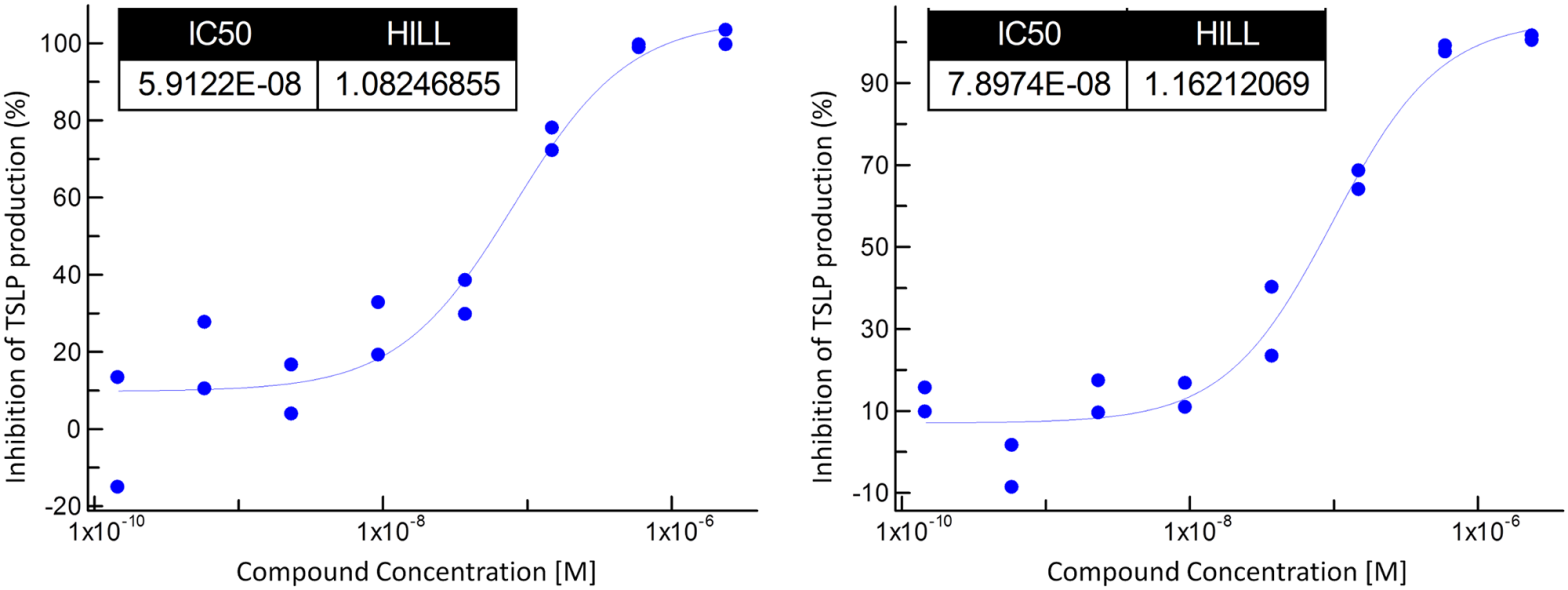
Activity of reference compound BX795 in the TSLP production assay. Activity in donors #1 (left panel) and #2 (right panel) is expressed as IC_50_ (M). Two representative curves are shown (n = 4 in each case).

The assay was validated using BX795, a non-selective kinase inhibitor which has been reported to downregulate dsRNA dependent TSLP production in bronchial epithelial cells from COPD donors [39] (Fig 1). BX795 targets within the pharmacological range (IC_50_ <1 μM) relevant in the context of this phenotypic assay include protein kinases TBK1/IKKɛ, PDK-1, Aurora B, ERK8, MARK, NUAK1, MLK, c-Kit, CDK2/CyclinE, Chk1, GSK-3β, PKA, KDR, T-Fyn and PCK [26].

### Primary phenotypic screen

Given the limitations imposed by the finite source of human donor derived lung epithelial cells, a subset of 44,974 compounds from the Almirall compound collection was selected for the phenotypic screen, as described in the Materials & Methods section. This relied on selecting plates with the best possible balance of the 4 criteria which were considered more relevant in the context of this screen: structural diversity, physicochemical properties compatible with traversing the cell membrane, lack of structural alerts and low cytotoxicity prediction.

**Fig 2:**
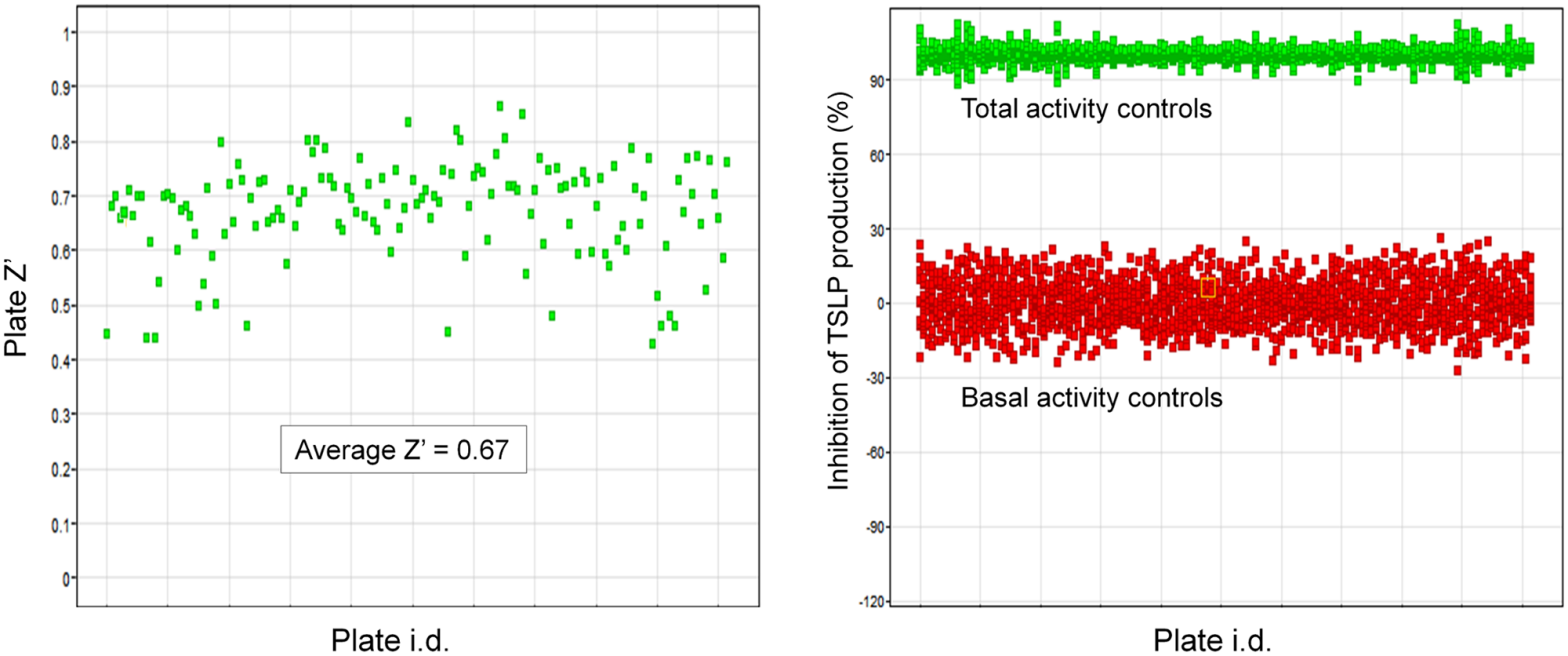
Quality control of primary phenotypic screen. (A) Z’ factor versus plate number. (B) Global representation of total and basal activity values for all plates.

Cells from donor #1 were used for the primary screen which was performed at a compound concentration of 1 μM. TSLP levels were quantified after 24h in the presence of test compounds as described in the Materials & Methods section. Quality control parameters are shown in Fig 2.

**Fig 3:**
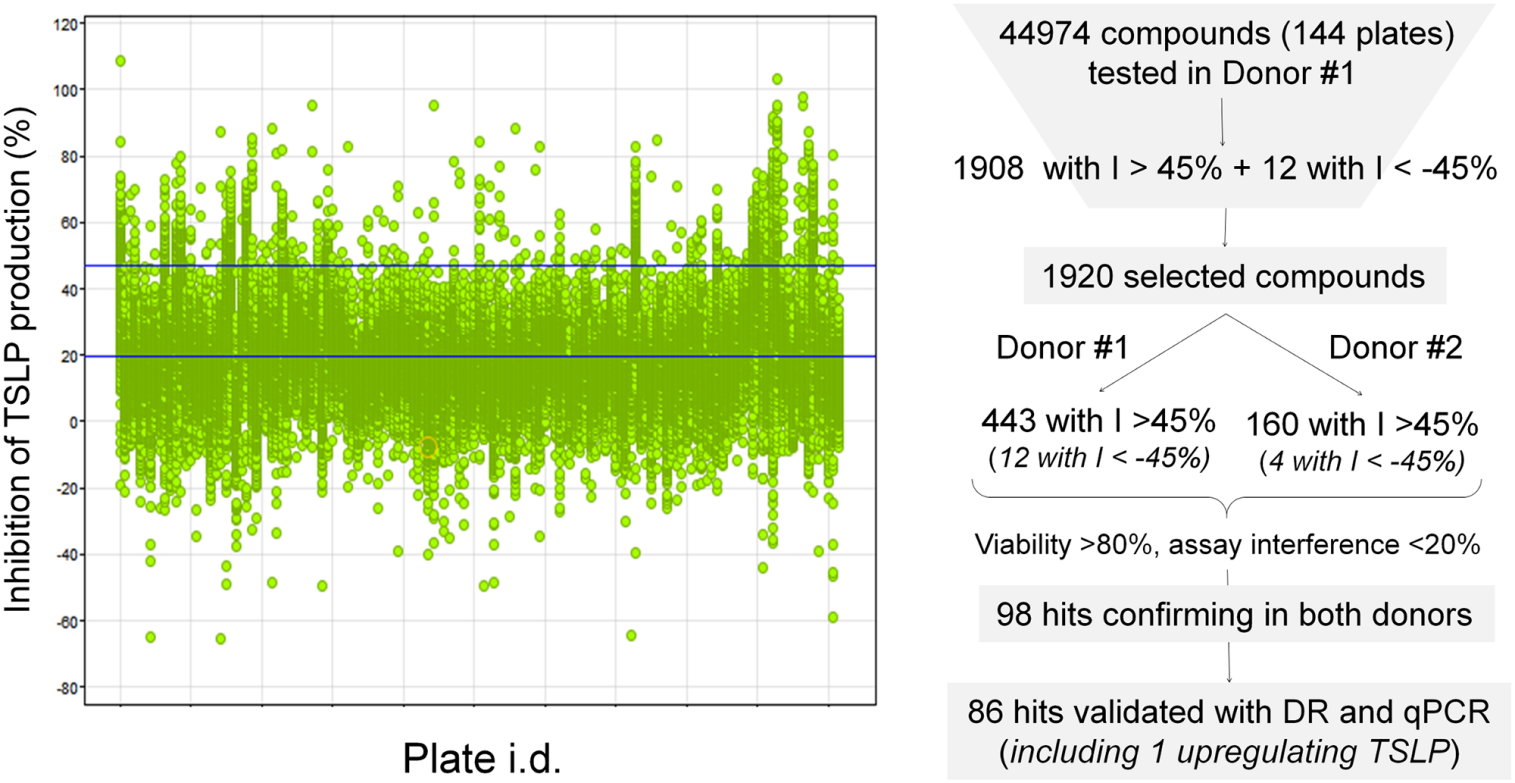
Overview of the screening and confirmation process. Left panel shows % inhibition of TSLP production (vertical axis) for all screened compounds distributed by plates (horizontal axis). Upper horizontal blue line in the left panel indicates cut off value for hit selection (45%). Right panel is a summary of the hit selection process (DR = dose response evaluation, I = inhibition).

Average Z’ factor was 0.67, and ranged between 0.45 and 0.85. Cut off inhibition for hit selection was 45%, just below the mean % inhibition + 2 standard deviations (SD) benchmark, which was 47%. This resulted in the identification of 1,908 compounds with > 45% inhibition of TSLP production. In addition, as a serendipitous finding, 12 compounds that were inducers of TSLP production were also identified. In total, 1,920 compounds were selected as output from the primary phenotypic screen (Fig 3).

To confirm and validate the candidate hits obtained in the primary screen, three additional assays were performed in NHBE cells: single concentration confirmation and dose response evaluation in the TSLP production assay, and single concentration determination of TSLP mRNA levels.

Hit confirmation was performed using cells from donors #1 and #2 and the set of 1,920 active compounds identified in the primary screen. Of the 1,920 initial hits, 443 confirmed their activity (>45% inhibition of TSLP production) in cells from donor #1, and 160 in cells from donor #2 (Fig 3). Among these, a common subset of 120 hits showed activity >45% in cells from both donors, with the highest coincidence between donors seen in the most active compounds: 77 out of the 80 most potent hits confirmed activity in donors #1 and #2 simultaneously. All 12 compounds inducing TSLP production confirmed their activity in cells from donor #1. Of those, 4 were also active in cells from donor #2.

To discard any effect resulting from impaired cellular viability or interference with the technology used in the TSLP production assay, compounds having cytotoxicity levels >20% in NHBE cells from donor #1 and showing an assay interference >20% were discarded. In this manner, a set of 98 compounds was finally identified as the output of the confirmation screen.

Following confirmation, the set of 98 compounds was characterized performing dose response curves with cells from the 2 donors and a concentration range of up to 4 μM. All of the compounds showed a dose response in at least one of the donors.

**Fig 4:**
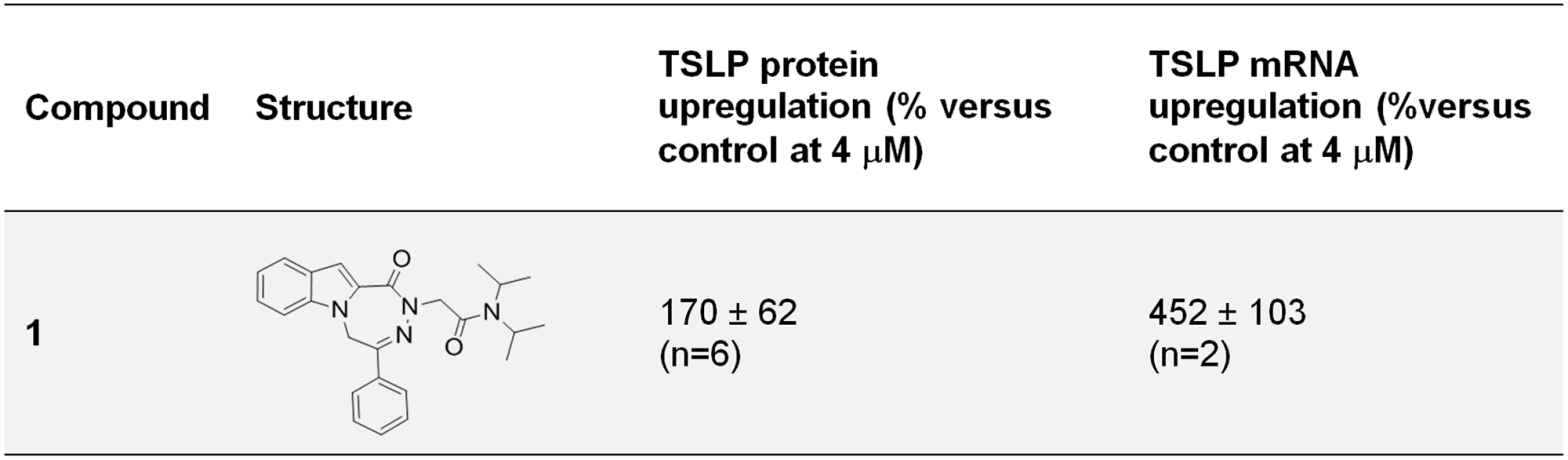
Upregulation of TSLP protein production and mRNA levels by compound 1. Results were obtained using NHBE cells from donor #1 and are shown as average % upregulation ± SD (n = number of replicates).

In parallel with the dose response and to further validate the hits using an alternative biological endpoint, the levels of TSLP mRNA were quantified using qPCR in cells from donor #1 after 6 h of poly I:C / IL-4 stimulation in the presence of test compounds. This provided an orthogonal assay that allowed ruling out any potential artifact related with the fluorescent HTRF antibody based technology used to measure TSLP protein levels. Results of this assay indicated a good correlation between TSLP protein and mRNA endpoints: 86 of the 98 hits showing a dose response were validated in the qPCR assay, 85 with inhibition of mRNA levels >45%, and compound **1**, one of the hits previously identified as inducer of TSLP protein levels, which was also found to increase mRNA levels (Fig 4).

### Hit analysis and selection

Given that the focus of this study was to identify inhibitors of TSLP production, compound **1** was not further characterized. *In silico* prediction of potential targets using the target prediction tool CT-Link [25] identified Neuropeptides B/W receptor Type 1 (NPBWR1), Translocator protein (TSPO) and Melanin concentrating hormone receptor 1 (MCHR1) as the top 3 predicted targets of this molecule.

**Fig 5:**
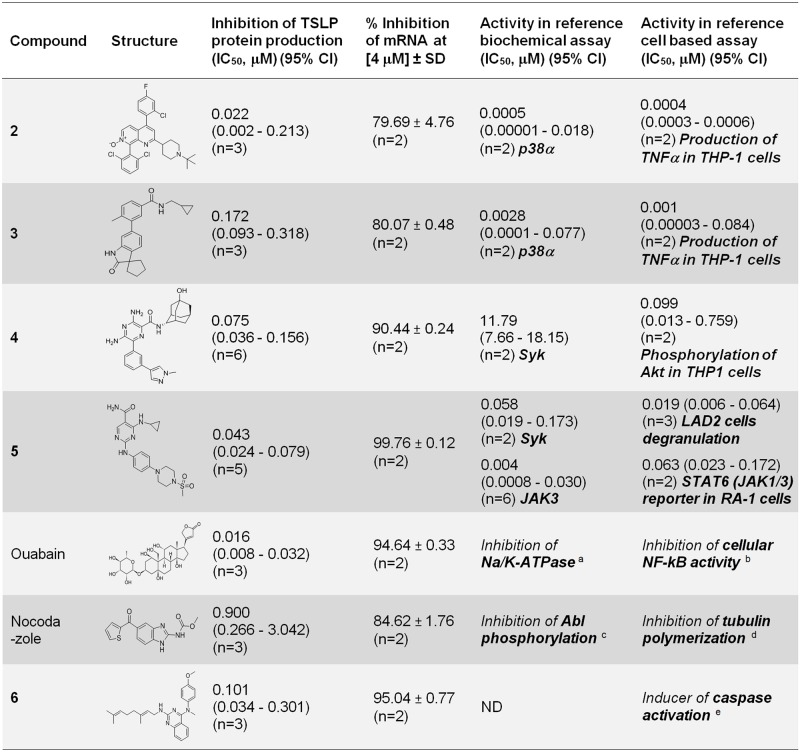
List of selected hit compounds. For TSLP protein and mRNA determinations results were obtained using NHBE cells from donor #1. Results are shown as geometric mean IC_50_ (μM) (95% confidence interval) or average % inhibition ± SD at the indicated concentration [μM]. Specific activities evaluated are indicated in each case and shown in bold. Activities reported in ^a^ [44], ^b^ [45], ^c^ [46], ^d^ [47] and ^e^ [32].

After analysis of the remaining 85 validated hits to remove compounds showing structural redundancy and containing structural alerts, a representative set of 7 confirmed and validated hits were selected for additional characterization. Of these, 3 hits were derived from in house programs aimed at the identification of inhibitors of p38 (compounds **2**, **3**) and Syk (compound **4**) kinases [40, 41, 42]. The 4 remaining hits were of external origin: compound **5** [43], ouabain [30], nocodazole [31] and compound **6** [32] (Fig 5).

An initial assessment of the hits derived from internal programs showed that compounds **2** and **3**, derived from a p38 program, were potent inhibitors of p38α, while compound **4**, derived from a Syk program was not an inhibitor of Syk (Fig 5). In the absence of a Syk dependent cellular activity, hit compound **4** was found to be active in a PI3K dependent cellular assay in M-SCF stimulated THP-1 cells (Fig 5).

The reported activities of the 4 external hits were as follows (Fig 5): (i) Compound **5**, is a potent Syk/JAK1, 2, 3 inhibitor reported as compound 99 in [43], (ii) Ouabain, a human endogenous hormone involved in the regulation of cell adhesion, is an inhibitor of NF-kB signaling and Na/K-ATPase that was initially identified as a plant derived cardiac glycoside [30, 44, 45, 48], (iii) Nocodazole, a microtubule polymerization inhibitor which targets Abl kinase, is also an inducer of the tumor suppressor LATS2 [31, 46, 47, 49], (iv) Compound **6**, has been described as a caspase activator [32].

### Hit profiling

**Fig 6:**
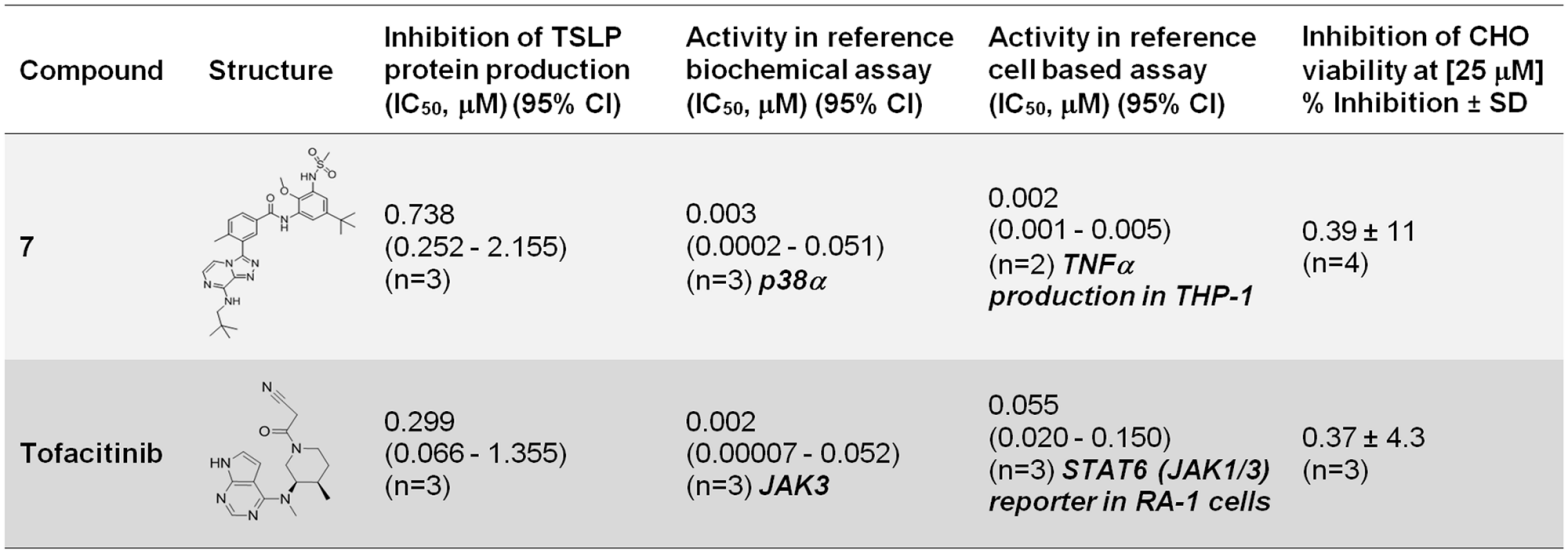
Correlation between inhibition of TSLP production and biochemical and cellular kinase activities of reference compound 7 (p38 inhibitor) and tofacitinib (JAK inhibitor). For TSLP protein determinations, results were obtained using NHBE cells from donor #1. Results are shown as geometric mean IC_50_ (μM) (95% confidence interval) or average % inhibition ± SD at the indicated concentration [μM]. Specific activities evaluated are indicated in each case and shown in bold.

In order to confirm the role of p38 and JAK kinases in the control of TSLP production in our phenotypic screening system, additional reference inhibitor compounds were evaluated: compound **7**, a selective p38 inhibitor that had not been evaluated in the primary phenotypic screen, and the selective JAK1/JAK2/JAK3 reference inhibitor tofacitinib [29] (Fig 6).

The activity of compound **7** in the TSLP NHBE assay was moderate (IC_50_ = 0.738 μM) and 369 fold less potent than the reference p38 dependent cellular activity of this compound (IC_50_ = 0.002 μM) (Fig 6). This ratio was 55 and 172 fold for hit compounds **2** and **3** respectively (Fig 5).

Evaluation of Tofacitinib showed a ratio of 5.4 fold between potency in the TSLP NHBE assay (IC_50_ = 0.299 μM) and reference JAK1/3 dependent cellular activity (IC_50_ = 0.055 μM) (Fig 6), while hit compound **5** showed comparable activities in both TSLP NHBE (IC_50_ = 0.043) and JAK1/3 dependent activity in the reference cellular assay (IC_50_ = 0.063) (Fig 5).

Given these different levels of correlation and in order to identify other potential activities that could be contributing to the inhibition of TSLP in addition to p38 and JAK kinases, compounds **2**, **3**, **4** and **5** were tested in a 125 kinase selectivity panel at a concentration of 10 μM (S3 Table). The profile of reference compound **7** was also tested in this panel to verify its selectivity for p38 MAP kinase. Results indicated that the hits derived from the in house p38 inhibitors program, compounds **2** and **3**, were very selective inhibitors of p38α(SAPK2α),β(SAPK2β) and MKK6 (inhibitions of ~ 100%) at this concentration. As secondary activities, compound **2** also inhibited TrkB (75%) and compound **3** inhibited Fms (53%). Both compounds showed an inhibition of less than 50% in the rest of the panel. Reference compound **7** also showed selectivity for p38α, β and MKK6 (Inhibition ~ 100%) with DDR2 (97%), Fms (74%), PTK5 (96%) and Tie2 (83%) as secondary activities (S3 Table).

Compound **4** showed a relatively selective profile inhibiting 9 out of the 125 kinases of the panel with a % inhibition > 50: TrkA (86%), Ftl4 (80%), Yes (77%), Lck (56%), DDR2 (53%), Hck (53%), PI3K family kinases mTOR (87%), PI3K (p120γ) (67%) and PI3K (p110δ/p85α) (79%). Compound **5**, showed multiple kinase inhibition activities at that concentration (>50% in 89 kinases), indicating that other activities in addition to JAK kinases could be contributing to the inhibition of TSLP production.

In the absence of a primary kinase target, and given its relatively selective profile, compound **4** was selected for further biological profiling in order to explore the mechanisms underlying the inhibition of TSLP production. Compound **4** was evaluated in a general selectivity panel (Bioprint panel, Eurofins) containing a broad representation of enzymes, ion channels and G protein coupled receptors and in a human primary cell based phenotypic profiling panel (BioMAP, DiscoverX) consisting of cells and co-cultures activated under a variety of stimulatory conditions. In both cases, the resulting bioactivity fingerprint was compared with a database containing drugs and reference molecules to provide hypotheses on mechanism of action and potential safety liabilities.

Results from the general selectivity panel screened at a concentration of 1 μM, indicated that there were 3 activities inhibited at >50%: MT3 (71%), PDE5 (61%) and PDE6 (55%) (S4 Table). Inhibition of 129 additional activities tested at this concentration was less than 50% (S4 Table). The resulting profile was then compared with Eurofins internal Bioprint database and no relevant similarity was identified (Pearson correlation 0.37–0.47).

**Fig 7:**
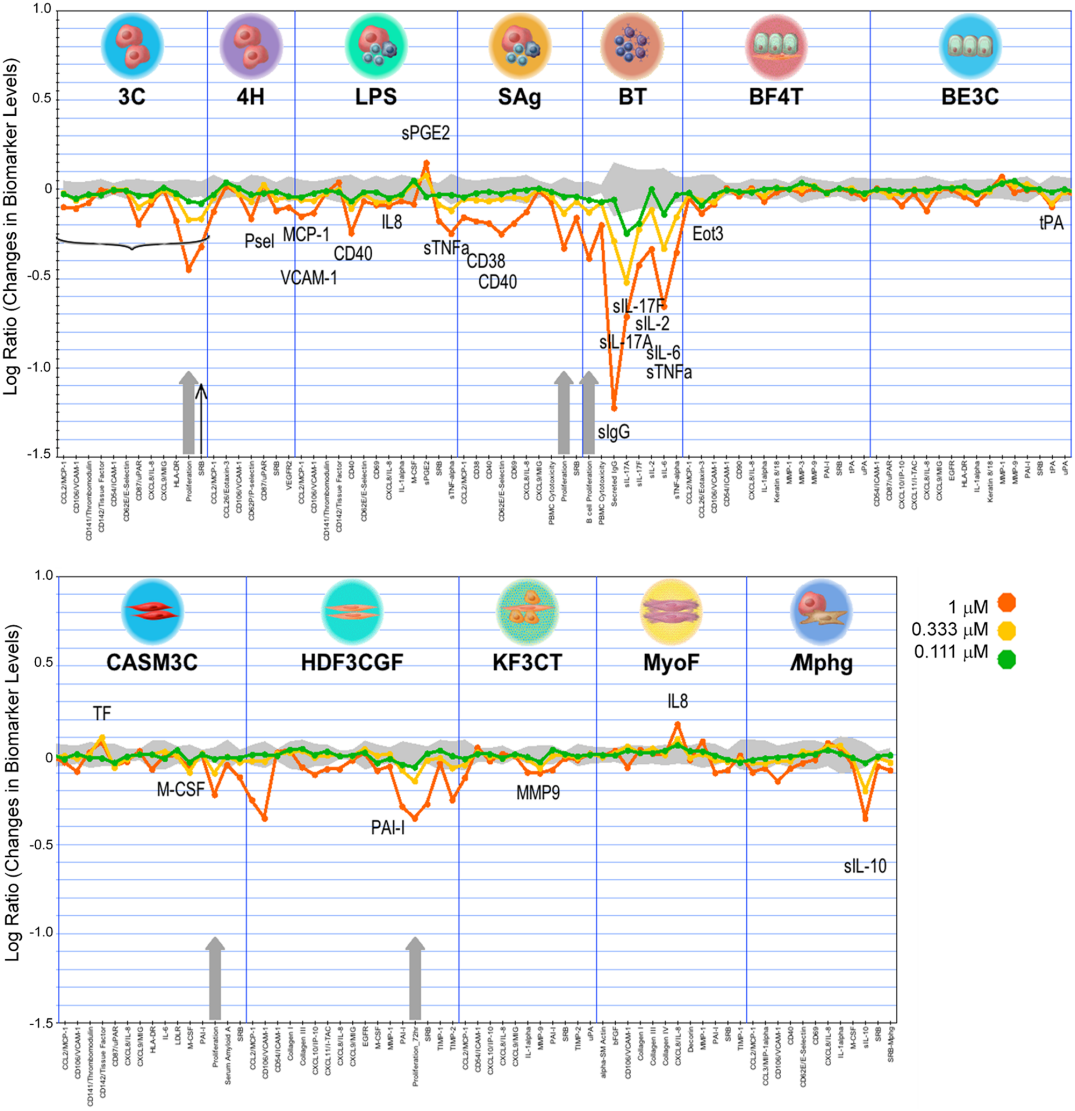
BioMAP activity profile of compound 4. Black thin arrow indicates cytotoxicity at 1 μM in the 3C system. The 3C system was excluded from further analysis at this concentration (indicated by horizontal bracket). Grey arrows indicate anti-proliferative activity. Abbreviations (full details in Materials & methods and S2 Table): 3C and 4H (HuVEC, venular endothelial cells), LPS and SAg (PBMC, peripheral blood mononuclear cells + HuVEC), BT (B cells + PBMC), BE3C (NHBE, normal human bronchial epithelial cells), BF4T (NHBE + NHDF, normal human dermal fibroblasts), HDF3CGF (NHDF), KF3CT (NHK, normal human keratinocytes + NHDF), CASM3C (CASMC, coronary artery smooth muscle cells), MyoF (HLF, human lung fibroblasts) and /Mphg (HuVEC, M1 macrophages).

In the phenotypic profiling BioMAP analysis, performed at 4 concentrations (0.111, 0.333, 1 and 3 μM) in 12 stimulated primary cell systems, the profile of compound **4** showed evidence of immunomodulatory and tissue remodeling activities (Fig 7, S2 Fig).

At the highest concentration tested (3 μM) compound **4** was cytotoxic to B cells, fibroblasts and particularly endothelial cells in these activated cellular systems (S2 Fig). At lower concentrations, 0.111 and 0.333 μM (Fig 7), it was anti-proliferative to endothelial cells, T and B cells, smooth muscle cells and fibroblasts, increased IL-8 levels in lung fibroblasts (MyoF), decreased sIL10 levels in the endothelial cell / macrophage system (/Mphg), reduced VCAM-1 expression in endothelial cells (3C) and decreased sIL17A, sIL17F and sIL6 levels in the B / PMBC cell system (BT) (Fig 7). These results also showed that phenotypic inhibition of TSLP production in NHBE cells was selective, since only 2 (tPA and Eotaxin 3) out of 32 endpoints measured in the NHBE (BE3C) and NHBE / fibroblasts (B4FT) systems were inhibited in the 0.111–0.333 μM range (Fig 7). Analysis by a similarity search of the BioMAP database using this profile identified a match with mTOR inhibitors everolimus [27] (Pearson correlation = 0.785) (S3 Fig) and temsirolimus [50] (S4 Fig) (Pearson correlation = 0.871) at concentrations of 0.333 μM and 1 μM of compound **4** respectively.

Based on the information obtained from the characterization of compound **4** in the BioMAP panel, the inhibition of TSLP production by reference mTOR inhibitors everolimus and AZD8055 [28] was evaluated. Both mTOR inhibitors proved to be potent blockers of TSLP production in the absence of relevant cytotoxicity (Table 3; S3 Fig).

**Table 3:** Inhibition of TSLP production by mTOR inhibitors everolimus and AZD8055.

Compound	Inhibition of TSLP protein production (IC_50_, μM) (95% CI) or % Inhibition ± SD at [μM]^a^	Inhibition of CHO viability at [3.12 μM] % Inhibition ± SD
Everolimus	79.4% ± 17.4 at 0.031 μM (n = 4)	-0.16% ± 2.3 (n = 4)
AZD8055	0.002 (0.0002–0.027) (n = 3)	20.47% ± 4.6 (n = 4)

Results shown as geometric mean IC_50_ (95% confidence interval) or average % inhibition ± SD at the indicated concentration (n = number of replicates).

^a^ Results obtained using NHBE cells from donor #1.

Interestingly, the inhibition of TSLP production by compound **4** (IC_50_ = 0.075 μM) was 12 fold more potent than its inhibition of mTOR biochemical activity (IC_50_ = 0.92 μM) (Table 4). Further characterization of compound **4** identified several activities with IC_50_ in the 0.2–1 μM range: PI3K, DNA-PK, MT3, PDE6 and PDE5 (Table 4), which can be considered as potential candidates to contribute to the TSLP blocking activity of compound **4**. However, inhibition of TSLP production in NHBE cells remained as the most potent biological activity identified for this hit (IC_50_ = 0.075). The most relevant activities identified for compound **4** in the 0.2–5 μM range after evaluation of a total of more than 400 biochemical and functional cellular endpoints are shown in Table 4.

**Table 4:** Main pharmacological activities identified in compound 4.

Activity	Inhibition of indicated activity (IC_50_, μM) ^a^
TSLP production (NHBE)	0.075 ^b^
PI3K (p110δ/p85α)	0.233
DNA-PK	0.348
MT3 (QR2)	0.4
PDE6	0.58
PDE5	0.92
mTOR	0.964
Yes	2.386
Ftl4	3.132
TrkA	3.232
Hck	3.581
Lck	3.330
PI3K (p120γ)	4.782

^a^ Except for TSLP, all IC_50_ activities are based on biochemical assays dose response curves with duplicate determinations for each concentration.

^b^ Activity as indicated in Fig 5.

### Hit expansion

**Fig 8:**
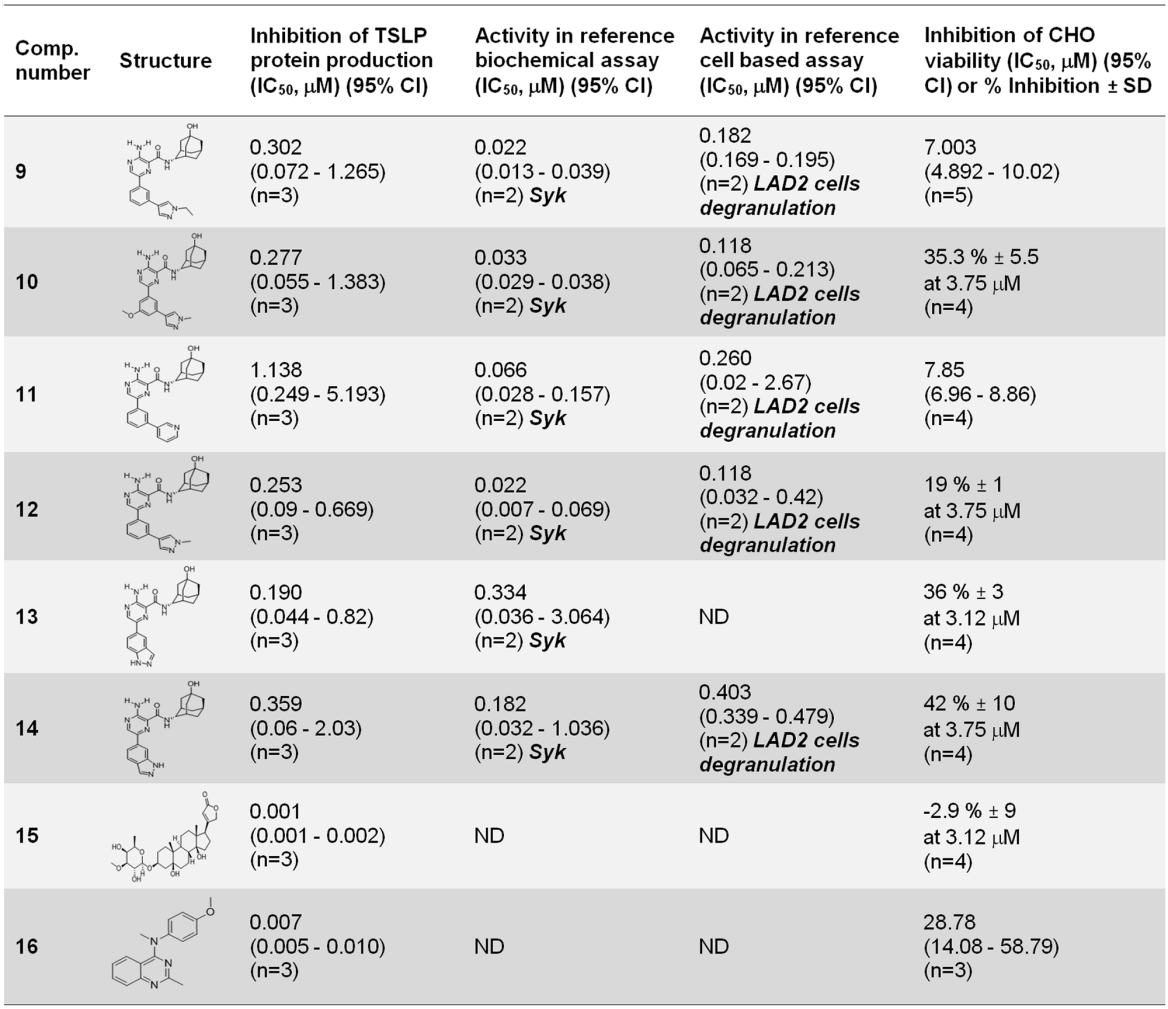
Active compounds identified in the hit expansion exercise. Results obtained using NHBE cells from donor #1 in the TSLP assay. Results shown as geometric mean IC_50_, μM (95% confidence interval) or average % inhibition ± SD at the indicated concentration. Biochemical and cell based specific activities evaluated are indicated in each case and shown in bold.

With the objective of assessing the potential for optimization of the selected hit compounds, a hit expansion campaign was undertaken using a set of compounds related to **4**, **5**, **6**, ouabain and nocodazole. Hit expansion offers the opportunity to dissociate TSLP blocking activity from other non-desired activities (e.g. microtubule disruption or caspase activation) by combining phenotypic based optimization and evaluation of activities identified in the biological profiling of the original compound. Using this approach, 6 compounds related to compound **4** were identified that showed inhibition of TSLP production in the range of IC_50_ = 0.1–1 μM: compounds **9–14** [42] (Fig 8).

Unlike compound **4**, all of them were inhibitors of Syk kinase. However, the presence of this activity did not result in an increase in TSLP production inhibition. This was also exemplified by comparing the profiles of compounds **11** and **14**: The increased biochemical and cellular Syk inhibition seen in compound **11** with respect to compound **14** did not result in increased inhibition of TSLP production (Fig 8).

Evaluation of compounds related with ouabain (IC_50_ = 0.016 μM in the TSLP assay) (Fig 5) yielded compound **15**, which showed a 16 fold increase in TSLP downregulation (Fig 8). Evaluation of compounds related to compound **6** (IC_50_ = 0.101 μM in the TSLP assay) (Fig 5) resulted in the identification of compound **16**, which showed a 14 fold increase in potency (IC_50_ = 0.007 μM) (Fig 8). Both compounds can be considered as potential chemical starting points for further profiling and optimization. No actives in the TSLP production assay were identified related to hit compound **5** and nocodazole.

## Discussion

A phenotypic drug discovery strategy provides a “jump start” opportunity of unbiased access to the targets or pathways driving the pathophysiology of disease, as long as these can be recapitulated by the selected *in vitro* model. For this reason the selection of a highly disease relevant cellular model is crucial for subsequent success in phenotypic drug discovery campaigns [15, 51].

**Fig 9:**
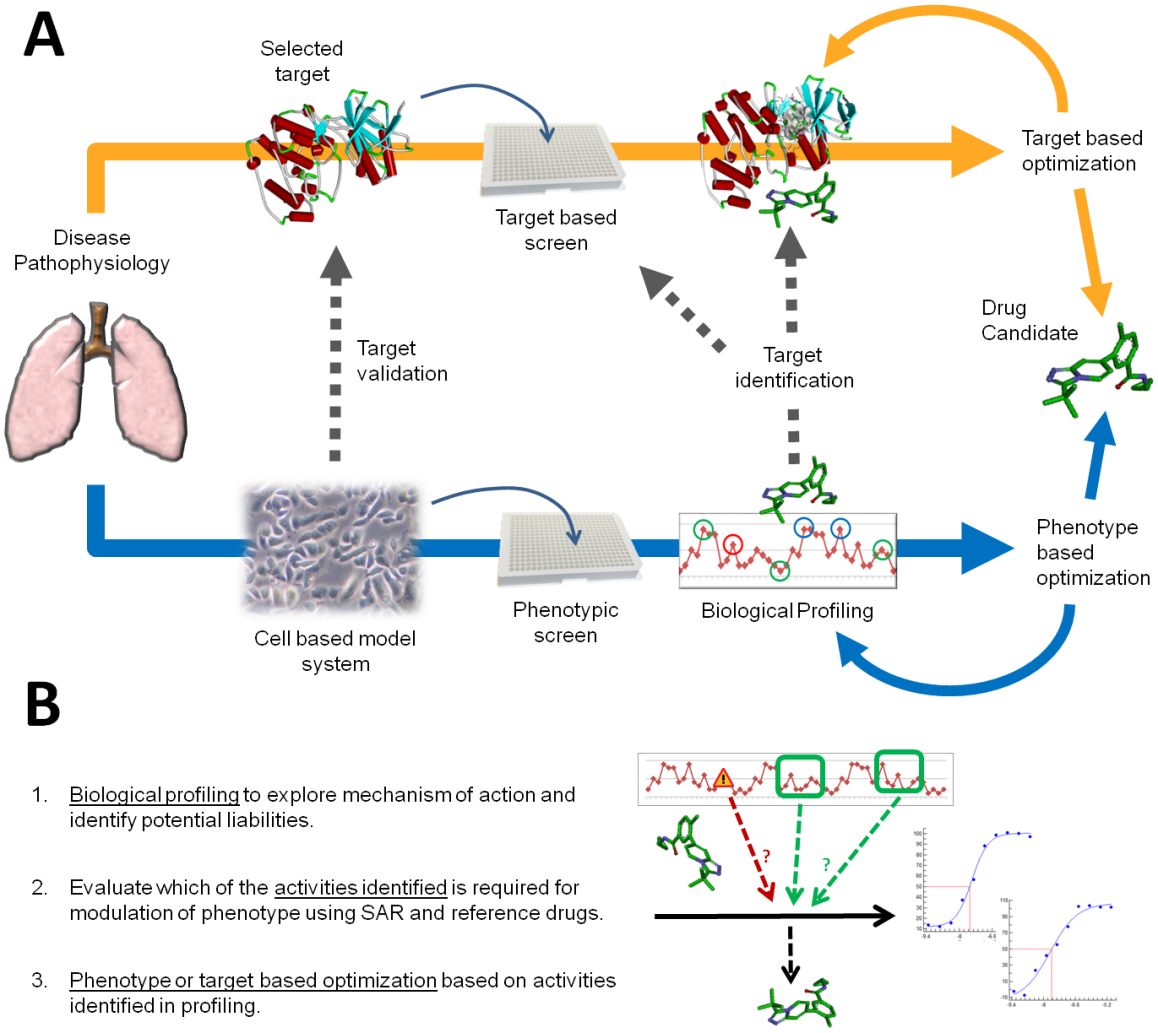
(A) Phenotypic and target based drug discovery strategies are complementary. (B) Phenotypic based optimization relies on biological profiling to identify both potential liabilities (represented in red) and targets responsible for activity (represented in green). SAR: structure—activity relationship.

Traditionally used in the pre-genomic era [52], phenotypic drug discovery has seen renewed interest in the last decade [12, 13, 14, 15] and is now consolidating its position as a powerful strategy that can be used in combination with the well proven target based approach [16]. Current technological advances in screening and biological profiling technologies and a more standardized and reliable access to human sourced samples now provide unique opportunities to fully exploit the approach. In the context of drug discovery, target and phenotypic based strategies are fully complementary and do not exclude each other (Fig 9A): target validation is based on functional phenotypic models, while phenotypic strategies can at any time divert to target based campaigns. Phenotypic screening can be seen as a non-biased gateway to identify the best possible strategy that can be used to engage the disease relevant endpoint of choice, be it target or phenotypic based.

In this study we have used human donor-derived primary lung epithelial cells in a 384 well plate format to evaluate a library of almost 45,000 compounds selected based on a balance between structural diversity, physicochemical properties compatible with intracellular targets, lack of structural alerts and low predicted cytotoxicity. The screen produced 86 hits which were confirmed in cells from 2 different human donors, verified for lack of cytotoxicity and validated measuring TSLP mRNA transcript levels. From this set, 1 compound was identified as an inducer of TSLP production, and 7 representative compounds downregulating TSLP levels were selected for further characterization. These hits represent a variety of biological and chemical starting points that could be used to develop new strategies to block TLSP production.

In order to set up a more physiologically relevant cellular system, the use of an air-liquid interface (ALI) system [53] to achieve differentiation of the bronchial epithelial cells was initially considered but was ultimately ruled out given the difficulties in achieving a high throughput format. A standard submerged culture format was finally adopted under the assumption that non-differentiated cells within the lamina propria also contribute to TLSP production in the lung [9].

The enrichment in kinase inhibitor hits seen in this screen may be in part due to the limitations of using target-focused libraries, many of which were initially designed to provide chemical starting points for kinase and GPCR inhibitor programs. This is an important limiting factor that should be taken into account when performing phenotypic screens. A phenotypic approach is an open strategy whose output will only be as good as the biological system and chemical library used in the screen.

The use of annotated or chemogenomic libraries offers a good tool to profile the biology of the model system being screened [54, 55] but, being based on previous knowledge, the approach falls short of providing the open chemical space that can allow full exploitation of phenotypic screens. On the other hand, using libraries based on maximum chemical diversity alone does not appear as a realistic option given the physicochemical restrictions imposed by a cell based assay with respect to intracellular targets, which would prevent taking full advantage of this approach. In contrast with the two previous approaches, enrichment in biologically active chemistry, identified by screening for perturbation of any measurable biological feature, may provide a good non biased criteria that can be used to build libraries that can better take advantage of the opportunities offered by a phenotypic screen [56].

Biological profiling of the hits obtained in this study indicated that they were inhibiting a variety of targets with different levels of selectivity. The targets were mostly MAPK, PI3K and Tyrosine family kinases (Abl, DNA-PK, JAK1-3, mTOR, p38 MAPK, PI3K, Syk, among others) but also included other proteins (MT3, PDE5, PDE6, Na/K-ATPase) and several phenotypic endpoints (NF-kB mediated signaling, microtubule polymerization, caspase activation, regulation of cell-cell contacts).

Analysis of the profile of compounds **9**–**14**, Syk inhibitors structurally similar to compound **4** (not an active Syk inhibitor) revealed that their TSLP blocking activity was not increased in spite of their capability to inhibit Syk, suggesting that this kinase was unlikely to be involved in the regulation of TSLP production (Fig 8).

Analysis of tofacitinib and reference compound **7**, potent and selective inhibitors of JAK and p38 / MKK6 kinases respectively, showed different levels of correlation between kinase inhibition and downregulation of TSLP production (Fig 6).

The profile of compound **7**, showing a 369 fold gap between TSLP and p38 cellular activities confirmed the lack of TSLP / p38 correlation seen in hit compounds **2** and **3** (55 and 172 fold ratios between TSLP and p38 cellular activities respectively), indicating that the inhibition of TSLP production seen in these compounds was unlikely to be directly related with their inhibition of p38 MAPK activity alone. However, a partial contribution of p38 in the context of a poly-pharmacological anti-TSLP production target footprint could not be ruled out. This is particularly intriguing for compounds **2** and **3** which were highly optimized compounds with good selectivity for p38 and MKK6. We speculate that, in this case, an additional non identified activity could be responsible for the inhibition of TSLP production, alone or in combination with p38 / MAPKK6. Considering their high level of optimization, direct target deconvolution may be a suitable alternative for identification of the targets involved in the inhibition of TSLP production in these compounds.

In contrast with reference compound **7** and hit compounds **2** and **3**, reference JAK inhibitor tofacitinib and hit compound **5** showed increased correlation in the TSLP and JAK dependent cellular assays. Tofacitinib showed a 5.4 fold drop in TSLP cellular potency with respect to JAK cellular potency while hit compound **5** showed very similar IC_50_ values between the two assays (Figs 5 and 6). These results are in agreement with previous evidence showing a role of JAK dependent STAT6 activation in IL-4-driven TSLP expression in NHBE cells [38]. However, given the poor kinase selectivity of hit compound **5** (S3 Table) it is very likely that other activities in addition to JAK inhibition may be contributing to its increased potency in the TSLP cellular assay with respect to tofacitinib (Figs 5 and 6).

The most clear evidence of the potential involvement of one of the targets identified during hit profiling in the control of TSLP production was obtained for mTOR, one of the kinases inhibited by compound **4** (IC_50_ ~ 1 μM) (Table 4). Evaluation of compound **4** in a primary human cell-based phenotypic profiling platform (the DiscoverX BioMAP Diversity PLUS panel) identified a match with mTOR inhibitors everolimus and temsirolimus (S3 and S4 Figs) which suggested the hypothesis that compound 4 could be engaging mTOR in the context of these activated cellular systems. Evaluation of everolimus and an additional mTOR reference inhibitor, AZD8055, in the TSLP / NHBE assay (Table 3), showed a downregulation of TSLP production that was comparable to their reported kinase inhibitory activity (IC_50_ ~0.001–0.002 μM) and even more potent than the reported reference cellular activity of AZD8055 (IC_50_ ~0.02 μM) [27, 28]. These results support a role of mTOR in the regulation of TSLP production in poly I:C / IL-4 stimulated NHBE cells. Nevertheless, inhibition of TSLP production by compound **4** in NHBE cells was seen at levels that do not fully correlate with mTOR inhibition, approximately 12 fold less potent in this compound (Table 4). This suggests that, even in this case, where a direct correlation between mTOR and the TSLP phenotype appears likely, a more complex poly-pharmacological profile combining one or more additional activities was probably driving the selective inhibition of TSLP production in NHBE cells in addition to mTOR. Table 4 lists 12 activities identified in compound **4** in the range of IC_50_ 0.2–5 μM. Systematic evaluation of reference inhibitors of these activities in the TSLP production assay would allow to identify or rule out potential targets involved in the regulation of TSLP production in addition to mTOR.

A role of mTOR in the onset of asthma has been proposed [57], and both the mTOR pathway and NF-kB are activated following TNFα/ IL-4 mediated stimulation in the bronchial epithelial cell line BEAS-2B [58]. Given that NF-kB is involved in the poly I:C dependent upregulation of TSLP in NHBE cells [11], our results now provide additional evidence to support a role of the mTOR / NF-kB axis in the upregulation of TSLP in NHBE cells after poly I:C / IL-4 stimulation. This is based on the pharmacological profile of compound **4**, everolimus and AZD 8055 and is further supported by the profile of external hits: ouabain has been reported to downregulate the TNFα / NF-kB pathway [45], while the tumour suppressor LATS2, upregulated by nocodazole [49], has also been reported to inhibit NF-kB mediated signaling [59].

Taken together these results indicate different levels of correlation between inhibition of mTOR, p38MAPK or JAK kinases, and TSLP downregulation, with none of these targets appearing sufficient on their own to explain the activity of the hit molecules, regardless of their relevance. The involvement of additional signaling pathways in the downregulation of TSLP production in addition to NF-kB / IRF-3 (poly I:C dependent) and JAK / STAT6 (IL-4 dependent) is not surprising, given the synergy observed when poly I:C and IL-4 are used in combination, which may lead to activation of other pathways not activated separately by the 2 stimuli [38].

In the context of phenotypic drug discovery, selectivity profiling studies can be used to support optimization through the identification of activities both required and not required to modulate the phenotype (Fig 9B). Based on that information, target and off target based optimization can be combined with phenotypic endpoint based optimization selecting one or the other option at any time during compound progression (Fig 9A). For highly optimized compounds direct target identification / deconvolution approaches may be justified to obtain conclusive evidence on the mechanism of action.

This screen allowed the serendipitous identification of several “hits” that were inducing TSLP protein and mRNA levels. One of these molecules, compound **1**, was confirmed and validated in cells from donors #1 and #2. An *in silico* target prediction study identified NPBWR1, TSPO and MCHR1 proteins as the top 3 potential targets of this compound. Interestingly TSPO, a mitochondrial transport protein, has been suggested as a potential target for asthma in children, based on protein-protein interaction network predictions supported by differential expression analysis of expression profiling data [60]. Further pharmacological characterization of this compound may provide additional insight into the mechanisms controlling TSLP mRNA and protein levels.

A hit expansion exercise to identify more potent compounds similar to compound **4** confirmed the structure—activity relationship of this chemical series but did not identify more active inhibitors of TSLP production (Fig 8). Further profiling of these compounds based on the activities identified in compound **4** (Table 4) could be used to pinpoint the targets that contribute to maintain TSLP blocking activity, complementing the evidence obtained using reference compounds.

Interestingly, evaluation of compounds similar to ouabain and compound **6** resulted in the identification of compounds **15** and **16** which were 16 and 14 fold more potent than their parent compounds blocking TSLP production respectively (Figs 5 and 8). These 2 molecules could also provide valuable starting points that could be used to explore the possibility to dissociate the TSLP phenotypic activity from their original activities (Na/K-ATPase inhibition and caspase activation).

Overall, our results illustrate the wealth of biological and chemical data that results from approaching an otherwise conceptually target based screen (inhibition of TSLP activity) with a phenotypic strategy (inhibition of TSLP production), providing chemical and biological starting points that can be used for the identification of new inhibitors of TSLP production in the future. This work is an example of how target and phenotypic based strategies can be combined to maximize the efficiency of the early drug discovery process, applying both approaches with the ultimate goal of providing new innovative clinical drug candidates.

## Supporting information

S1 Table Individual data values Tables 1 and 3, Figs 1, 4, 5, 6 and 8 and GADPH controls in qPCR. (XLSX)

S2 Table Details of BioMAP^®^ cell systems. Cell types, stimuli and evaluated biological endpoints.
(XLSX)

S3 Table Evaluation of compounds in the kinase selectivity panel. Values show percentage of control activity (POC) at a concentration of 10 μM of test compound. Highlighted in darker grey activities < 50%. Values shown are the mean of 2 replicate determinations.
(XLSX)

S4 Table Evaluation of compound 4 in the Bioprint^®^ selectivity panel. Values show percentage of inhibition of the indicated activity at a concentration of 1 μM of test compound (10 μM for P-gp and CYP enzymes). Highlighted in darker grey inhibitions > 50%. Values shown are the mean of 2 replicate determinations. INTER = assay interference.
(XLSX)

S1 Fig Detection of TSLP mRNA by endpoint PCR in NHBE cells. M: DNA marker; NS: non stimulated cells; IL-4: cells stimulated 50 ng/mL IL-4; IL-4 + poly I:C: cells stimulated with 10 μg/mL poly I:C and 50 ng/mL IL-4 as indicated.
(TIF)

S2 Fig Full BioMAP profile of Compound 4 at the indicated concentrations. Thin black arrows indicate cytotoxicity seen at the top 3 μM concentration (3 and 1 μM for HUVEC 3C cells). Grey arrows indicate inhibition of proliferation seen in the 3C, Sag, BT, CASM3C and HDF3CGF systems. Full details of the model systems can be found in S2 Table.
(TIF)

S3 Fig BioMAP database match of compound 4 with everolimus. For compound **4**, thin black arrows indicate cytotoxicity and grey arrows indicate inhibition of proliferation. Full details of the model systems can be found in S2 Table.
(TIF)

S4 Fig BioMAP database match of compound 4 with temsirolimus. For compound **4**, thin black arrows indicate cytotoxicity and grey arrows indicate inhibition of proliferation. Full details of the model systems can be found in S2 Table.
(TIF)
