# Supplementary figures and images for: Application of a phenotypic drug discovery strategy to identify biological and chemical starting points for inhibition of TSLP production in lung epithelial cells
Source: PLoS One. 2018 Jan 10;13(1):e0189247. doi: 10.1371/journal.pone.0189247 (PMC5761851; doi:10.1371/journal.pone.0189247)

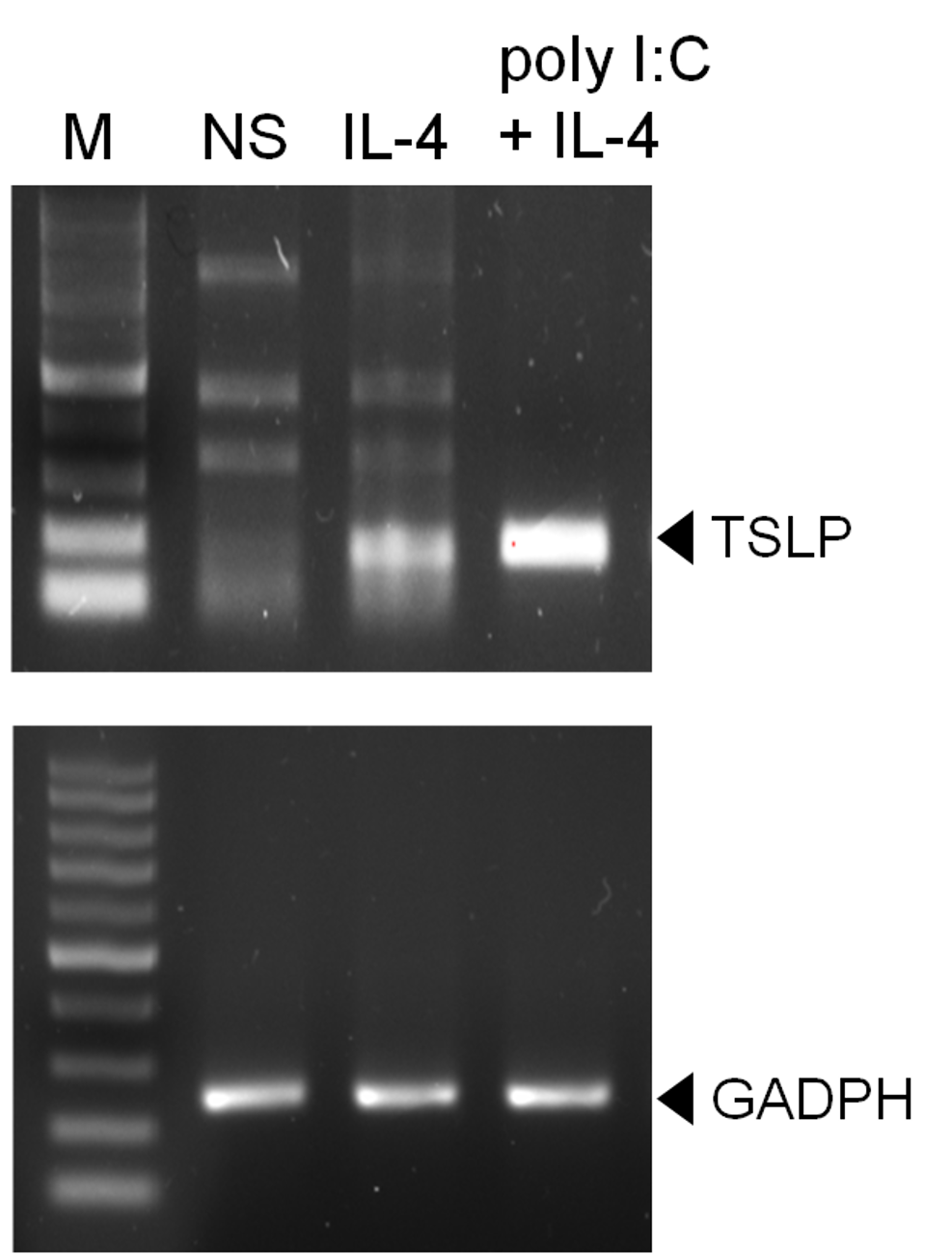

Supplement: S1 Fig — M: DNA marker; NS: non stimulated cells; IL-4: cells stimulated 50 ng/mL IL-4; IL-4 + poly I:C: cells stimulated with 10 μg/mL poly I:C and 50 ng/mL IL-4 as indicated. (TIF) [file pone.0189247.s005.tif]

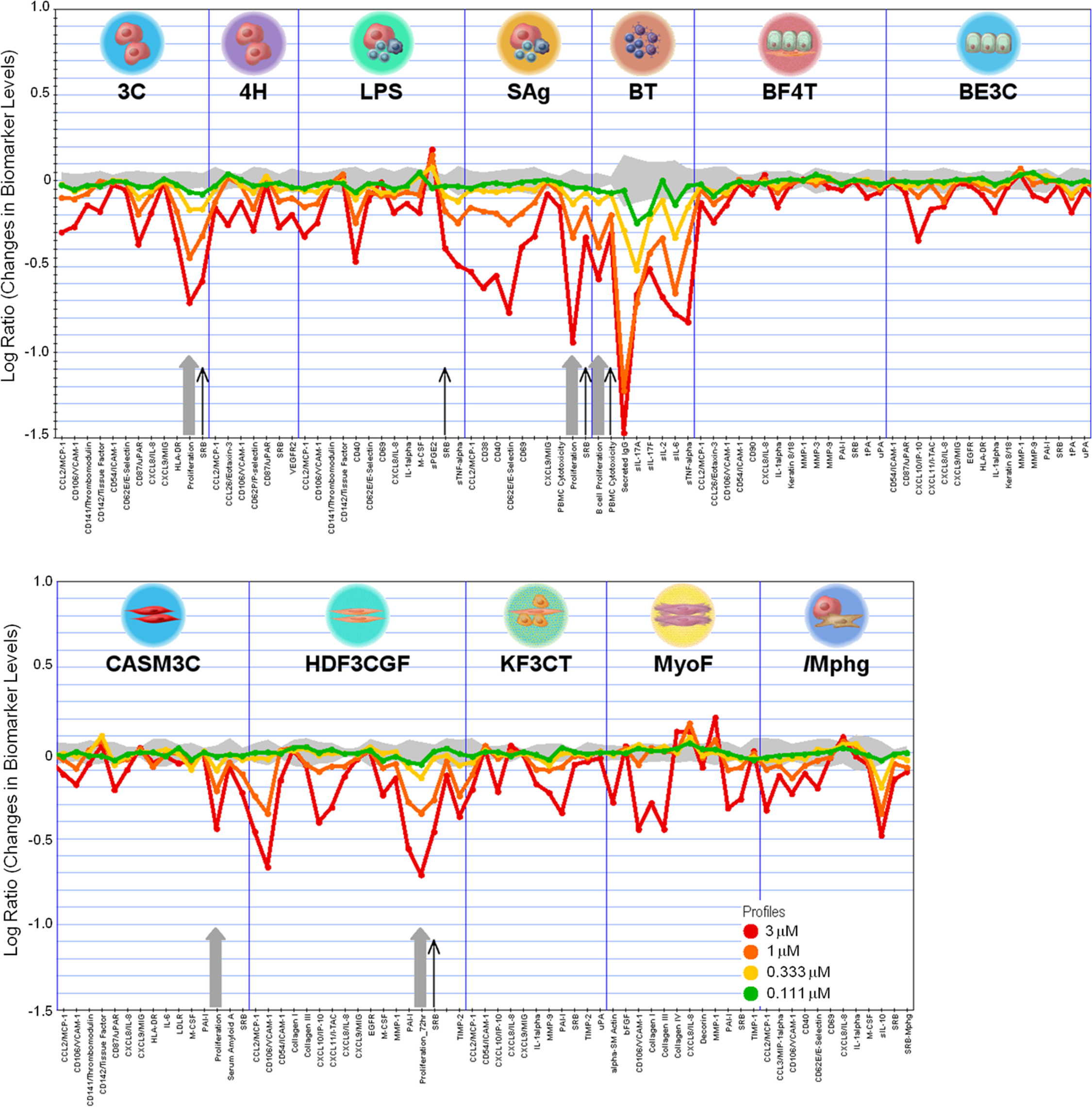

Supplement: S2 Fig — Thin black arrows indicate cytotoxicity seen at the top 3 μM concentration (3 and 1 μM for HUVEC 3C cells). Grey arrows indicate inhibition of proliferation seen in the 3C, Sag, BT, CASM3C and HDF3CGF systems. Full details of the model systems can be found in S2 Table. (TIF) [file pone.0189247.s006.tif]

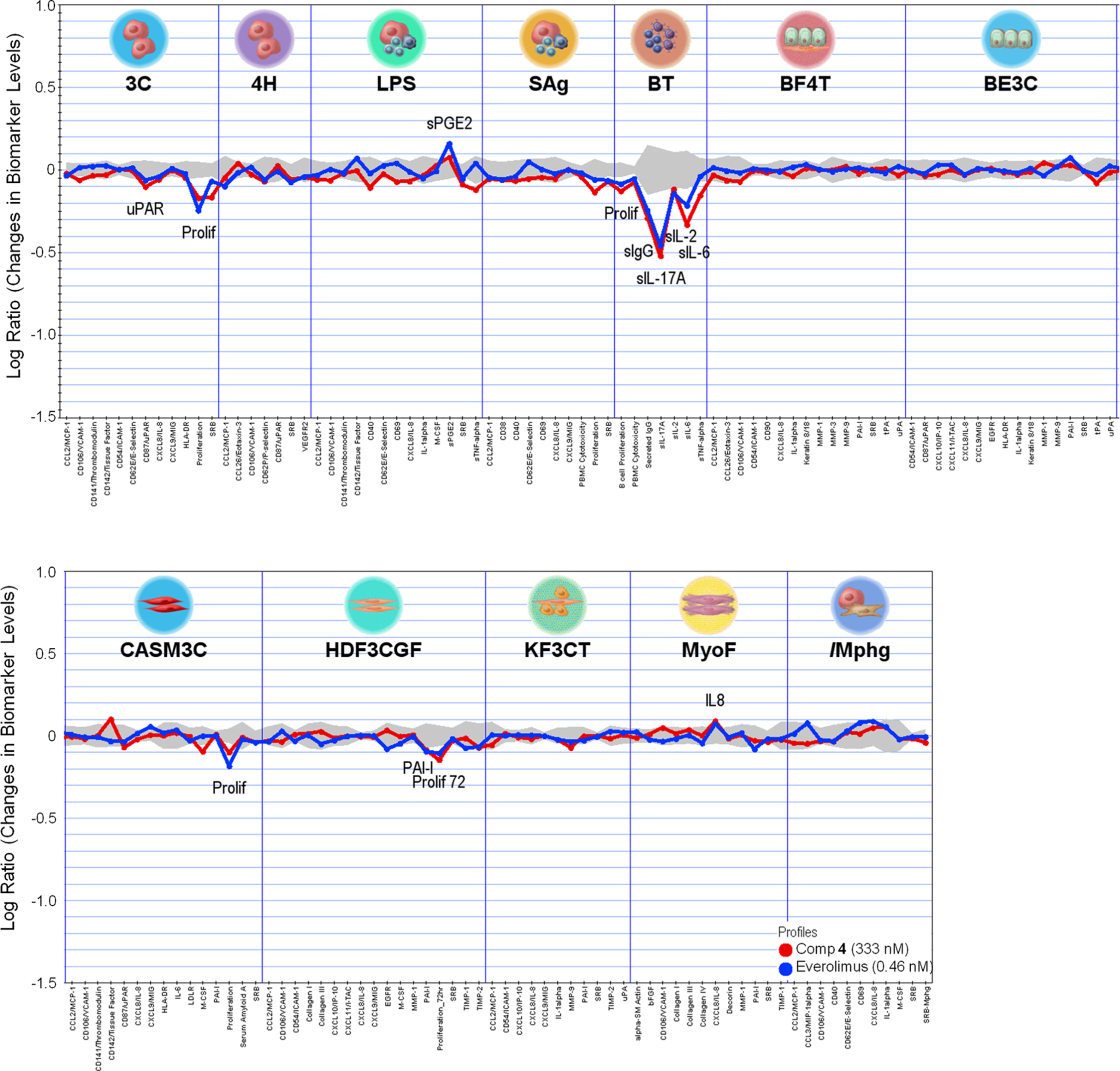

Supplement: S3 Fig — For compound 4, thin black arrows indicate cytotoxicity and grey arrows indicate inhibition of proliferation. Full details of the model systems can be found in S2 Table. (TIF) [file pone.0189247.s007.tif]

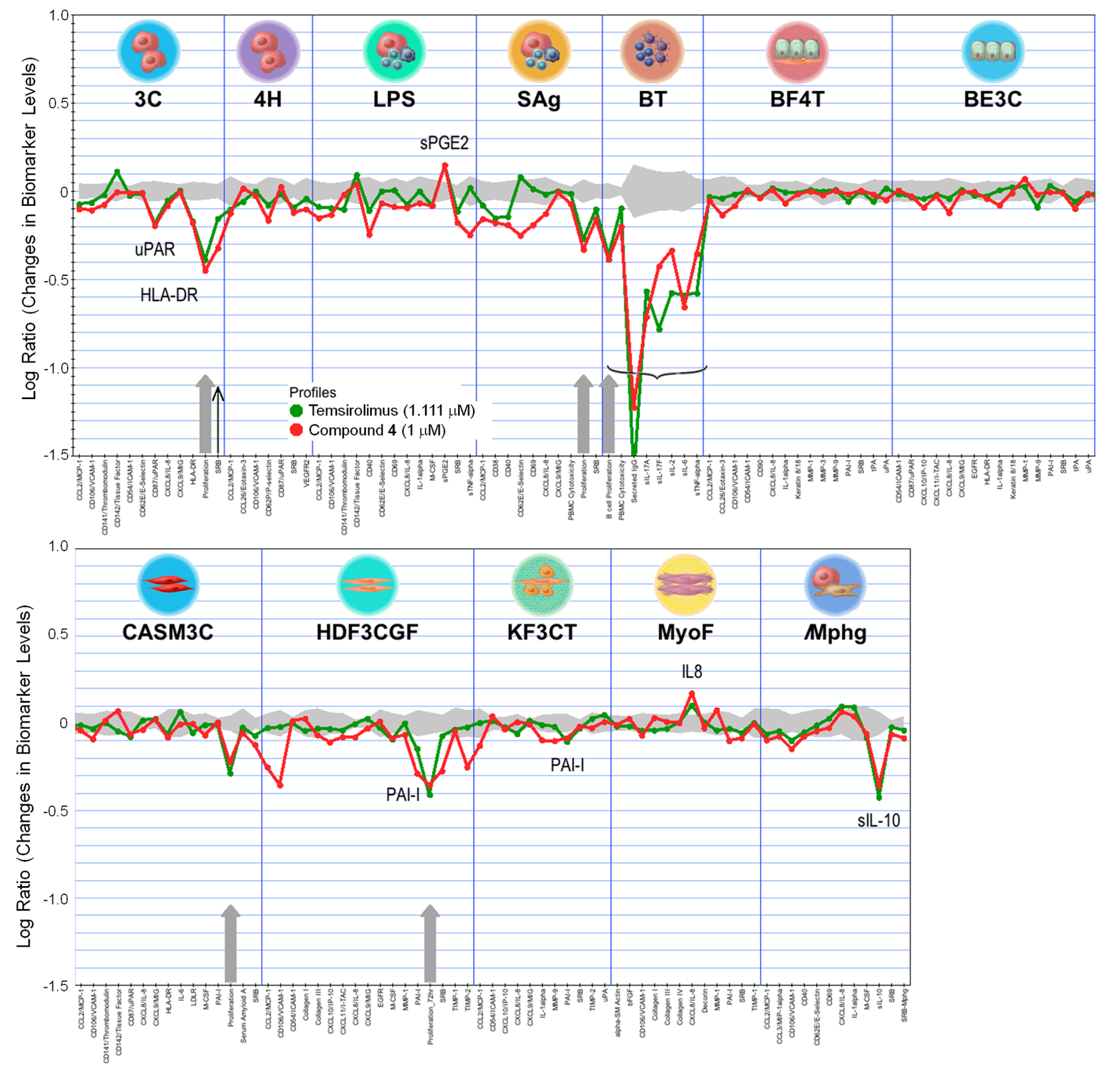

Supplement: S4 Fig — For compound 4, thin black arrows indicate cytotoxicity and grey arrows indicate inhibition of proliferation. Full details of the model systems can be found in S2 Table. (TIF) [file pone.0189247.s008.tif]
